# Effect of the Age-Adjusted Charlson Comorbidity Index on All-Cause Mortality and Readmission in Older Surgical Patients: A National Multicenter, Prospective Cohort Study

**DOI:** 10.3389/fmed.2022.896451

**Published:** 2022-06-28

**Authors:** Xiao-Ming Zhang, Xin-Juan Wu, Jing Cao, Na Guo, Hai-Xin Bo, Yu-Fen Ma, Jing Jiao, Chen Zhu

**Affiliations:** Department of Nursing, Chinese Academy of Medical Sciences-Peking Union Medical College, Peking Union Medical College Hospital, Beijing, China

**Keywords:** older adults, surgical patients, mortality, readmission, age-adjusted Charlson comorbidity index

## Abstract

**Background:**

Identifying a high-risk group of older people before surgical procedures is very important. The study aimed to explore the association between the age-adjusted Charlson comorbidity index (ACCI) and all-cause mortality and readmission among older Chinese surgical patients (age ≥65 years).

**Methods:**

A large-scale cohort study was performed in 25 general public hospitals from six different geographic regions of China. Trained registered nurses gathered data on clinical and sociodemographic characteristics. All-cause mortality was recorded when patients died during hospitalization or during the 90-day follow-up period. Readmission was also tracked from hospital discharge to the 90-day follow-up. The ACCI, in assessing comorbidities, was categorized into two groups (≥5 vs. <5). A multiple regression model was used to examine the association between the ACCI and all-cause mortality and readmission.

**Results:**

There were 3,911 older surgical patients (mean = 72.46, SD = 6.22) in our study, with 1,934 (49.45%) males. The average ACCI score was 4.77 (SD = 1.99), and all-cause mortality was 2.51% (high ACCI = 5.06% vs. low ACCI = 0.66%, *P* < 0.001). After controlling for all potential confounders, the ACCI score was an independent risk factor for 90-day hospital readmission (OR = 1.18, 95% CI: 1.14, 1.23) and 90-day all-cause mortality (OR = 1.26, 95% CI: 1.16–1.36). Furthermore, older surgical patients with a high ACCI (≥5) had an increased risk of all-cause mortality (OR = 6.13, 95% CI: 3.17, 11.85) and readmission (OR = 2.13, 95% CI: 1.78, 2.56) compared to those with a low ACCI (<5). The discrimination performance of the ACCI was moderate for mortality (AUC:0.758, 95% CI: 0.715–0.80; specificity = 0.591, sensitivity = 0.846) but poor for readmission (AUC: 0.627, 95% CI: 0.605–0.648; specificity = 0.620; sensitivity = 0.590).

**Conclusions:**

The ACCI is an independent risk factor for all-cause mortality and hospital readmission among older Chinese surgical patients and could be a potential risk assessment tool to stratify high-risk older patients for surgical procedures.

## Introduction

With the broad expansion of health science and economics, people are experiencing increased longevity, resulting in an aging society around the world (1). It is predicted that the percentage of older people aged 65 years or older will exceed 16% of the global population by 2050 (2). On the other hand, the demand for healthcare for older adults is sharply rising (3), and over 50% of surgical operations are for patients older than 65 (4). It is estimated that over 75% of older adults suffer from at least one chronic disease, and most of them benefit from surgical treatment for ailments such as cancer, fractures, or coronary artery disease. Meanwhile, older people are a vulnerable group due to age-related decline in terms of physiological reserve and functional capacity, concurring with frequent comorbidities (5), which brings about a unique postoperative challenge and even unacceptable mortality (6). A previous study reported that the mortality rate among older people who underwent surgical procedures was higher than that among younger people (7). Therefore, clinical staff need to perform appropriate preoperative risk stratification and determine whether the benefits of surgical intervention outweigh the risks among older adults, which could help to educate and allow for informed patient consent.

There are several predictive tools for hospital mortality, such as the Simplified Acute Physiology Score and acute physiology and chronic health evaluations, producing good results for critically ill patients (8, 9). However, these predictive tools are not appropriately applied in hospitalized older patients before surgery. Other predictive tools, such as the American Society of Anesthesiologists (ASA) classification system, are based on subjective assessments of illness in patients, thereby limiting their potential clinical application (10). The Charlson comorbidity index (CCI), developed in 1987, has been widely applied in surgical patients to evaluate the impact of comorbidity status on patient outcomes (11). Previous studies have shown that patients with a higher CCI have an increased risk of adverse outcomes such as mortality, extended hospital stays or hospital readmission (12–14). Since age was reported to influence patient survival, in 1994, Charlson et al. modified the CCI by adding age to the scoring system. Therefore, the age-adjusted Charlson comorbidity index (ACCI) incorporated age as an adjusted variable of the final scoring system. The ACCI has better performance for predicting adverse mortality than the CCI (15).

The age-adjusted Charlson Comorbidity Index has been reported to be associated with a high risk of readmission and mortality in older surgical patients (16, 17). A study by Gatot et al. (16) al revealed that the rate of 90-day mortality was significantly higher among older surgical patients with a higher ACCI score (≥6) than among those with an ACCI score of 0-3. Other studies also found similar results that higher ACCI scores could predict short-term or long-term outcomes (18, 19). Few studies have explored the association between the CCI and adverse outcomes among older Chinese surgical patients (20, 21). However, there remains a paucity of articles examining the impact of ACCI for forecasting negative outcomes in older Chinese patients based on a large-scale study. The aim of our study was to scrutinize the association between ACCI and adverse outcomes, such as mortality and hospital readmission, among older Chinese surgical patients based on a national cohort study. We hypothesize that surgical patients with a higher ACCI are at higher risk of mortality or hospital readmission among older Chinese adults.

## Methods

### Design: This is a National Multicenter, Prospective Cohort Study

This national multicenter prospective cohort study originated from a nationwide nursing project designed to evaluate the effect of a standardized nursing care program for improving the quality of nursing for bedridden patients to reduce the rate of major complications, such as pressure injuries, deep vein thrombosis (DVT), urinary tract infection and pneumonia (22).

### Participants and Sample

**Figure 1:**
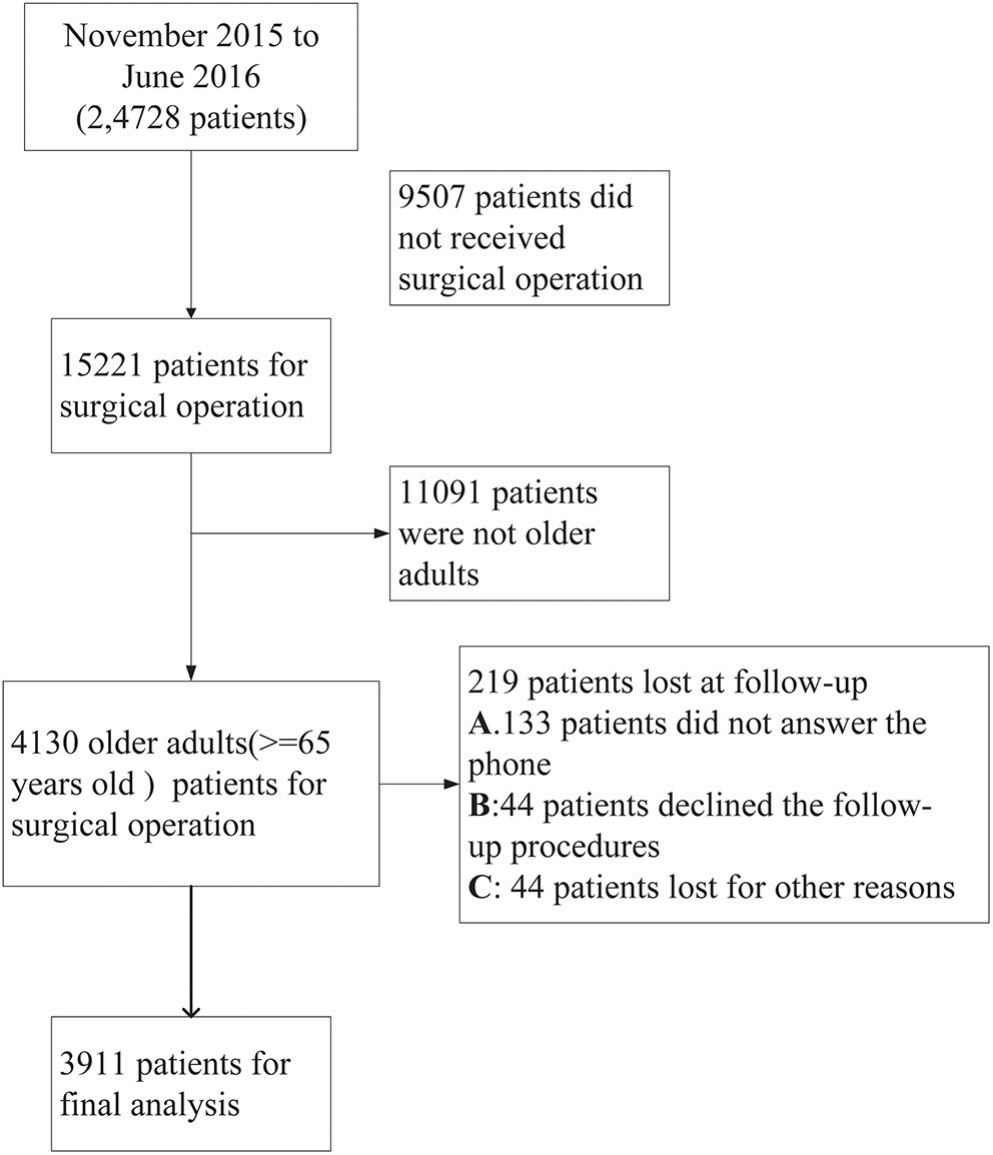
Flow chart of the study population selection including patient recruitment, exclusion criteria.

This is a larger-scale cohort study conducted in 25 general public hospitals from six different geographic regions of China (Beijing, Zhejiang Province, Guangdong Province, Hubei Province, Henan Province, and Sichuan Province). A baseline survey was administered between November 2015 and March 2016. A two-stage cluster sampling method was adopted for recruiting eligible participants admitted to medical or surgical and critical care units. According to the sample size calculation formula for categorical data: N = K×Q/P, K is equal to 400 when considering a two-sided 95% confidence interval with a width of 0.1. The prevalence of pressure injury (P) was 1.577%, and Q was equal to 1-P. Therefore, 24,600 patients need to be investigated after applying this sample size calculation formula. In the original study, there were 24,728 patients at the baseline survey, and we excluded 9,507 who did not receive surgical treatment, resulting in 15,221 patients. After deleting those young adults, 4,130 older adults aged 65 years older or above were included. However, there were 219 patients lost at the 90-day follow-up, of whom 133 patients did not answer the phone, 44 patients declined the follow-up procedure, and 44 patients were lost for other reasons. Finally, there were 3,911 older adult surgical patients included in our final analysis (Figure 1). Patients who met the inclusion and exclusion criteria were recruited into the original study. The original inclusion criteria were as follows: (1) aged ≥ 18 years old; (2) staying in bed for more than 24 h after the onset of admission; and (3) agreed to participate in this study and signed the informed consent form. In addition, the exclusion criteria were as follows. (1) Patients who had already experienced four major complications (pressure injuries, DVT, urinary tract infection and pneumonia) were excluded; and (4) those who had undergone unconscious conditions were excluded. Further information about this study can be found elsewhere (23). The Ethics Committee of Peking Union Medical Hospital approved this study (#S-700). For the present study, we extracted data from older adults aged 65 years or above who had undergone surgical operations.

### Data Collection

According to our previous research (23), 787 registered nurses from selective hospitals were responsible for data collection. Initially, an electronic data collection system that covered all the contents of the Case Report Form (CRF), including demographic data and related health variables, was created for collection and management data. Registered nurses screened patients and confirmed the available patients after hospital admission once they met the inclusion and exclusion criteria. Then, registered nurses finished all the items of CRF within 48 h after recruitment. To control the quality of the collection data, a standard training program for the data collection procedure was provided to these appointed registered nurses to completely understand the entire study, including the design, aim, and inclusion and exclusion criteria. Furthermore, they acquired the ability to use the electronic system to record raw data. Next, these team workers implemented a strict process for quality control. The head nurse of every selected department was responsible for checking and reviewing the data in case of any errors. In addition, the chief-in-charge managers of the program regularly held conferences to gather feedback and to identify problems to guarantee the quality of the data. For the follow-up procedure, patients received a telephone call by registered nurses to collect related information on health outcomes at 90 days after enrollment.

### The Covariates and ACCI

The covariates consisted of demographic features and clinical variables. Age, sex, education, ethnicity, smoking history, and body mass index (BMI; weight/height^2^) were collected. Furthermore, routine whole blood tests (such as white blood cells, red blood cells, hemoglobin, neutrophils, and leukomonocytes) were collected by perusing the medical records of patients' 48-h hospital admission. Other important variables included major complications (such as urinary tract infections [UTIs], pneumonia, deep vein thrombosis (DVT), pressure injury that occurred during hospitalization and urinary catheters, experiences of mechanical ventilation, and coma). Comorbidity was assessed using the CCI through the hospital electronic medical system, which has been widely reported. Medical staff reviewed the patients' clinical history and recorded existing comorbidities. The ACCI calculated 17 categories of comorbidity scores and considered age scores. A free online calculator was displayed (https://www.mdcalc.com/charlson-comorbidity-index-cci). In our study, we categorized ACCI into two groups (≥5 vs. <5) according to a previous study (24).

### Definition of Adverse Outcomes

All-cause mortality comprised two types of patients: those who died in the hospital or during the period of follow-up at 90 days. In addition, data on hospital readmission were gathered by phone 90 days after hospital discharge.

### Statistical Analyses

Descriptive analysis was employed to summarize the sample characteristics. Categorical and continuous data are presented as the mean ± standard deviation and frequency, respectively, adopting Student's *t* test and the chi-square test to identify the differences between different groups (ACCI ≥5 vs. <5, and survival vs. deceased). Multiple linear regression analysis and logistic regression analysis were used to detect the association between ACCI or categorical ACCI and adverse outcomes, respectively, such as all-cause mortality and hospital readmission. We defined potential confounding factors that were both related to dependent and independent factors. Therefore, in the present study, gender, age, BMI, smoking, pneumonia, coma, Braden socre, urinary catheters, mechanical ventilation, hemoglobin and leukomonocytes were potential confounding factors. We adjusted for these potential confounding factors to detect the independent relationship between the ACCI and death or hospital readmission. In addition, multicollinearity diagnosis showed no significant multicollinearity in our logistic regression analysis (Supplementary Table 1). We listed three models for adjusted variables: (1) Model 1 for unadjustment; (2) Model 2 adjusted for gender and age; and (3) Model 3 adjusted for gender, age, BMI, smoking, pneumonia, coma, Braden, urinary catheters, mechanical ventilation, hemoglobin and leukomonocytes. According to a previous study (25), patients over 75 years of age were defined as “much older.” Therefore, subgroup analysis of gender and age groups (≥75 vs. <75) was also performed between ACCI and adverse outcomes. ROC curve analysis was used to discern the performance of the ACCI for all-cause mortality and hospital readmission. In our total sample, the only variable of the Braden scale score had 139 (3.37%) missing data, which was <5%; therefore, we neglected the effect of this missing data. We used the statistical software packages R and Empowerstats software to scrutinize the data, revealing a significant difference of a two-sided *P*-value < 0.05.

## Results

### Baseline Characteristics

Table 1 summarizes the clinical and sociodemographic attributes among the total sample and the two different ACCI groups (≥5 vs. <5). There were 3,911 patients aged 65 or older who had undergone surgery, with an average age of 72.46 (SD = 6.22). The shares of females and males were similar (49.45% for males and 50.55% for females). The average ACCI of these samples was 4.77 (SD = 1.99), and the proportion of patients with high ACCI scores (ACCI ≥ 5) was 1641 (41.95%). In addition, the average BMI was 23.67 (SD = 3.58). Of these samples, nearly half of the patients had only a primary school degree or lower (51.60%). The prevalence of complications such as pressure injury, DVT, pneumonia, and UTIs was 3.86%, 2.81%, 7.41%, and 0.69%, respectively. Among this sample, 98 (2.51%) patients died, and 18.90% experienced hospital readmission. In general, the high ACCI group (≥5) suffered from a significantly higher percentage of pneumonia, coma, 90-mortality, and hospital readmission than the low ACCI group. Furthermore, there were significant differences between these two ACCI groups in terms of age, BMI, hemoglobin, white blood cells, neutrophils, leukomonocytes, gender, education, ethnicity, smoking, pressure injury, urinary catheters, and mechanical ventilation. However, other variables, such as UTI, DVT, and red blood cells, did not show any significant differences between the two ACCI groups.

**Table 1:** Baseline characteristics (Overall and ACCI group).

			**ACCI**	**P-value**
**Variables**	**All sample** **Mean ±SD or *n*%**	** <5** **Mean ±SD or *n*%**	**≥5** **Mean ±SD or *n*%**	
*N*	3,911	2,270	1,641	
Braden score	15.90 ± 2.26	16.25 ± 2.00	15.41 ± 2.50	<0.001
Age	72.46 ± 6.22	71.67 ± 6.18	73.55 ± 6.12	<0.001
BMI (kg/cm^2^)	23.67 ± 3.58	23.89 ± 3.64	23.38 ± 3.48	<0.001
Red blood cell (10^∧9^/L)	3.90 ± 1.32	3.90 ± 1.31	3.91 ± 1.35	0.872
White blood cell (10^∧9^/L)	10.13 ± 7.95	9.77 ± 4.61	10.64 ± 10.99	<0.001
Hemoglobin (g/L)	116.44 ± 19.85	117.12 ± 19.26	115.48 ± 20.60	0.012
Neutrophils 10^9^/L	8.09 ± 4.38	7.83 ± 4.36	8.44 ± 4.38	<0.001
Leukomonocyte (10^9^/L)	1.14 ± 0.76	1.18 ± 0.70	1.09 ± 0.85	<0.001
ACCL score	4.77 ± 1.99	3.58 ± 0.49	6.41 ± 2.11	<0.001
Gender				<0.001
Male	1,934 (49.45%)	996 (43.88%)	938 (57.16%)	
female	1,977 (50.55%)	1,274 (56.12%)	703 (42.84%)	
Ethnicity				0.002
Han	3,828 (97.88%)	2,208 (97.27%)	1620 (98.72%)	
Others	83 (2.12%)	62 (2.73%)	21 (1.28%)	
Education				<0.001
Primary school or lower	2,018 (51.60%)	1,242 (54.71%)	776 (47.29%)	
Middle school	903 (23.09%)	516 (22.73%)	387 (23.58%)	
High school	522 (13.35%)	281 (12.38%)	241 (14.69%)	
College/university	468 (11.97%)	231 (10.18%)	237 (14.44%)	
Smoking				<0.001
Never	3,029 (77.45%))	1,835 (80.84%)	1,194 (72.76%)	
Current	457 (11.68%)	252 (11.10%)	205 (12.49%)	
Former	425 (10.87%)	183 (8.06%)	242 (14.75%)	
Pressure injury				0.004
No	3,760 (96.14%)	2,165 (95.37%)	1,595 (97.20%)	
Yes	151 (3.86%)	105 (4.63%)	46 (2.80%)	
DVT				0.868
No	3,801 (97.19%)	2,207 (97.22%)	1,594 (97.14%)	
Yes	110 (2.81%)	63 (2.78%)	47 (2.86%)	
Pneumonia				<0.001
No	3,621 (92.59%)	2,181 (96.08%)	1,440 (87.75%)	
Yes	290 (7.41%)	89 (3.92%)	201 (12.25%)	
Urinary tract infection				0.068
No	3884 (99.31%)	2,259 (99.52%)	1,625 (99.02%)	
Yes	27 (0.69%)	11 (0.48%)	16 (0.98%)	
Coma				<0.001
No	3,669 (93.81%)	2,182 (96.12%)	1,487 (90.62%)	
Yes	242 (6.19%)	88 (3.88%)	154 (9.38%)	
Mechanical Ventilation				<0.001
No	3,260 (83.35%)	1,982 (87.31%)	1,278 (77.88%)	
Yes	651 (16.65%)	288 (12.69%)	363 (22.12%)	
Urinary catheters				
No	1,159 (29.63%)	786 (34.63%)	373 (22.73%)	<0.001
Yes	2752 (70.37%)	1,484 (65.37%)	1,268 (77.27%)	
90-day hospital readmission				<0.001
No	3,172 (81.10%)	1,967 (86.65%)	1,205 (73.43%)	
Yes	739 (18.90%)	303 (13.35%)	436 (26.57%)	
90-day mortality				<0.001
No	3,813 (97.49%)	2,255 (99.34%)	1,558 (94.94%)	
Yes	98 (2.51%)	15 (0.66%)	83 (5.06%)	
General anesthesia				0.088
No	677 (17.31%)	373 (16.43%)	304 (18.53%)	
Yes	3,234 (82.69%)	1,897 (83.57%)	1,337 (81.47%)	

*ACCI, Age adjusted Charlson Comorbidity Index; DVT, Deep vein thrombosis*.

### Univariate Analysis for the Association Between Variables and All-Cause Mortality or Hospital Readmission

Tables 2, 3 outline the association between variables and death or hospital readmission. Overall, age, pressure injury, pneumonia, coma, mechanical ventilation, ACCI score, and urinary catheters were risk factors for mortality (all *P* < 0.05). Being female, red blood cell count, hemoglobin, BMI, and Braden score were protective factors for mortality (all *P* < 0.05). In addition, age, white blood cells, neutrophils, smoking, pressure injury, pneumonia, coma, DVT, mechanical ventilation, ACCI socre, and urinary catheters were risk factors for hospital readmission (all *P* < 0.05). However, other variables, such as female sex, leukomonocyte count, BMI, and Braden score, were protective factors for increasing hospital readmission (all *P* < 0.05).

**Table 2:** Univariate analysis for 90-day mortality.

	**90-day mortality**		
**Variables**	**No**	**Yes**	***P*-value**
*N*	3,813	98	
Age	72.38 ± 6.18	75.72 ± 7.00	<0.001
Red blood cell (10^∧^9/L)	3.91 ± 1.33	3.52 ± 0.78	0.004
Hemoglobin (g/L)	116.71 ± 19.68	105.76 ± 23.37	<0.001
White blood cell (10^∧^9/L)	10.13 ± 8.02	10.31 ± 4.33	0.823
Neutrophils10^∧^9/L	8.08 ± 4.38	8.49 ± 4.42	0.366
Leukomonocyte (10^∧^9/L)	1.14 ± 0.75	1.09 ± 1.20	0.507
BMI	23.71 ± 3.58	22.16 ± 3.31	<0.001
Braden score	15.95 ± 2.23	13.93 ± 2.83	<0.001
ACCI	4.72 ± 1.96	6.45 ± 2.41	<0.001
Gender			0.006
Male	1,872 (49.10%)	62 (63.27%)	
Female	1,941 (50.90%)	36 (36.73%)	
Ethnicity			0.140
Han	3,730 (97.82%)	98 (100.00%)	
Others	83 (2.18%)	0 (0.00%)	
Education			0.072
Primary school or lower	1,955 (51.27%)	63 (64.29%)	
Middle school	884 (23.18%)	19 (19.39%)	
High school	513 (13.45%)	9 (9.18%)	
College/university	461 (12.09%)	7 (7.14%)	
Smoking			0.618
Never	2,957 (77.55%)	72 (73.47%)	
Current	443 (11.62%)	14 (14.29%)	
Former	413 (10.83%)	12 (12.24%)	
Pressure injury			0.025
No	3,670 (96.25%)	90 (91.84%)	
Yes	143 (3.75%)	8 (8.16%)	
Pneumonia			<0.001
No	3,550 (93.10%)	71 (72.45%)	
Yes	263 (6.90%)	27 (27.55%)	
Urinary tract infection			0.102
No	3,788 (99.34%)	96 (97.96%)	
Yes	25 (0.66%)	2 (2.04%)	
Coma			<0.001
No	3,596 (94.31%)	73 (74.49%)	
Yes	217 (5.69%)	25 (25.51%)	
DVT			0.880
No	3,706 (97.19%)	95 (96.94%)	
Yes	107 (2.81%)	3 (3.06%)	
Mechanical Ventilation			<0.001
No	3,194 (83.77%)	66 (67.35%)	
Yes	619 (16.23%)	32 (32.65%)	
ACCI group			<0.001
<5	2,255 (59.14%)	15 (15.31%)	
≥5	1,558 (40.86%)	83 (84.69%)	
Urinary Catheters			<0.001
No	1,145 (30.03%)	14 (14.29%)	
Yes	2,668 (69.97%)	84 (85.71%)	
General anesthesia			0.275
No	656 (17.20%)	21 (21.43%)	
Yes	3,157 (82.80%)	77 (78.57%)	

*ACCI, Age adjusted Charlson Comorbidity Index; DVT, Deep vein thrombosis*.

**Table 3:** Univariate analysis for 90-day hospital readmission.

	**90-day hospital readmission**		***P*-value**
**Variables**	**No Mean ±SD**	**Yes** **Mean ±SD**	
	**or *n* %**	**or *n* %**	
N	3172	739	
Age	72.30 ± 6.05	73.16 ± 6.87	<0.001
Red blood cell (10^∧^9/L)	3.91 ± 1.33	3.87 ± 1.28	0.420
Hemoglobin (g/L)	116.71 ± 19.49	115.28 ± 21.26	0.082
White blood cell (10^∧^/L)	10.08 ± 8.55	10.37 ± 4.55	0.003
Neutrophils (10^∧^9/L)	8.01 ± 4.38	8.41 ± 4.35	0.026
Leukomonocyte (10^∧^9/L)	1.16 ± 0.77	1.08 ± 0.72	0.014
BMI (kg/cm2)	23.75 ± 3.61	23.34 ± 3.44	0.005
Braden score	16.00 ± 2.20	15.45 ± 2.51	<0.001
ACCI	4.60 ± 1.84	5.47 ± 2.39	<0.001
Gender			0.020
Male	1,540 (48.55%)	394 (53.32%)	
Female	1,632 (51.45%)	345 (46.68%)	
Ethnicity			0.846
Han	3,104 (97.86%)	724 (97.97%)	
Others	68 (2.14%)	15 (2.03%)	
Education			0.123
Primary school or lower	1,666 (52.52%)	352 (47.63%)	
Middle school	719 (22.67%)	184 (24.90%)	
High school	416 (13.11%)	106 (14.34%)	
College/university	371 (11.70%)	97 (13.13%)	
Smoking			<0.001
Never	2,498 (78.75%)	531 (71.85%)	
Current	354 (11.16%)	103 (13.94%)	
Former	320 (10.09%)	105 (14.21%)	
Pressure injury			<0.001
No	3,077 (97.01%)	683 (92.42%)	
Yes	95 (2.99%)	56 (7.58%)	
Pneumonia			<0.001
No	2,986 (94.14%)	635 (85.93%)	
Yes	186 (5.86%)	104 (14.07%)	
Urinary tract infection			0.587
No	3,149 (99.27%)	735 (99.46%)	
Yes	23 (0.73%)	4 (0.54%)	
Coma			<0.001
No	3,024 (95.33%)	645 (87.28%)	
Yes	148 (4.67%)	94 (12.72%)	
DVT			0.042
No	3,091 (97.45%)	710 (96.08%)	
Yes	81 (2.55%)	29 (3.92%)	
Mechanical Ventilation			0.004
No	2,670 (84.17%)	590 (79.84%)	
Yes	502 (15.83%)	149 (20.16%)	
ACCI group			<0.001
<5	1,967 (62.01%)	303 (41.00%)	
≥5	1,205 (37.99%)	436 (59.00%)	
Urinary Catheters			<0.001
No	1,009 (31.81%)	150 (20.30%)	
Yes	2,163 (68.19%)	589 (79.70%)	
General anesthesia			0.086
No	565 (17.81%)	112 (15.16%)	
Yes	2,607 (82.19%)	627 (84.84%)	

*ACCI, Age adjusted Charlson Comorbidity Index; DVT, Deep vein thrombosis*.

### The Effect of the ACCI or High ACCI on Adverse Outcomes, Such as Hospital Readmission and All-Cause Mortality

The results of multivariate regression indicated that the ACCI score was a risk factor for mortality (OR = 1.30, 95% CI: 1.21–1.38) and hospital readmission (OR = 1.20, 95% CI: 1.16–1.25) in the unadjusted model. After adjusting for sociodemographic features (such as age and gender), the ACCI and high-ACCI groups were still risk factors for mortality and hospital readmission. Furthermore, after adjusting for the full potential confounding factors, the ACCI score and the high-ACCI groups remained independent risk factors for mortality or hospital readmission, with the OR of the ACCI score for mortality being 1.26 (95% CI: 1.16–1.36), that for the high-ACCI group being 6.13 (95% CI: 3.17–11.85), that for the ACCI score for hospital readmission being 1.18 (95% CI: 1.14–1.23) and that for the high-ACCI group for hospital readmission being 2.13 (95% CI: 1.78–2.56) (Table 4). In addition, the sex and age groups of the subgroup analysis revealed these associations to be unchanged (Table 5).

**Table 4:** Multivariate regression analysis of the association between ACCI/ACCI group and adverse outcome.

**Exposure**	**Non-adjusted OR/95%CI *P*-value**	**Adjust I** **OR/95%CI** ***P*-value**	**Adjust II OR/95%CI *P*-value**
**90-day hospital readmission**			
ACCL score (per 1)	1.20 (1.16, 1.25) <0.0001	1.19 (1.15, 1.24) <0.0001	1.18 (1.14, 1.23) <0.0001
**ACCL group**			
<5	1.0	1.0	1.0
≥5	2.35 (2.00, 2.77) <0.0001	2.26 (1.92, 2.67) <0.0001	2.13 (1.78, 2.56) <0.0001
**90-day mortality**			
ACCL score(per 1)	1.30 (1.21, 1.38) <0.0001	1.28 (1.19, 1.37) <0.0001	1.26 (1.16, 1.36) <0.0001
ACCL group			
<5	1.0	1.0	1.0
≥5	8.01 (4.60, 13.93) <0.0001	6.82 (3.90, 11.92) <0.0001	6.13 (3.17, 11.85) <0.0001

*ACCI, Age-Charlson Comorbidity Index. Non-adjusted model adjusted for: None Adjust I model adjusted for: Gender; Age. Adjust II model adjusted for: Gender; Age; BMI; smoking; Pneumonia; coma; Braden; Urinary Catheters; Mechanical Ventilation; Hemoglobin, Leukomonocyte*.

**Table 5:** Subgroup analysis of the association between ACCI/ACCI group and adverse outcome by gender and age group.

**Exposure (ACCI score)**	**90-day mortality OR/95%CI/P-value**	**90-day hospital readmission** **OR/95%CI/P-value**
**Subgroup analysis**		
Gender		
Male	1.22 (1.10, 1.35) < 0.0001	1.18 (1.12, 1.24) < 0.0001
Female	1.38 (1.21, 1.57) < 0.0001	1.19 (1.11, 1.26) < 0.0001
Age group		
65 ≤ age <75	1.31 (1.18, 1.46) < 0.0001	1.22 (1.16, 1.27) < 0.0001
≥75	1.21 (1.07, 1.37) 0.001	1.10 (1.02, 1.19) 0.005
**Exposure [ACCI group (<5 vs**. **≥5)]**	**90-day mortality OR/95%CI/P-value**	**90-day hospital readmission** **OR/95%CI/P-value**
**Subgroup analysis**		
Gender		
Male	4.89 (2.13, 11.23) < 0.001	2.32 (1.79, 3.00) < 0.001
Female	10.74 (3.53, 32.68) < 0.001	1.89 (1.45, 2.47) < 0.001
Age group		
65 ≤ age <75	5.57 (2.45, 12.62) < 0.001	2.26 (1.81, 2.82) < 0.001
≥75	10.26 (2.88, 36.58) < 0.001	1.89 (1.36, 2.62) < 0.001

*ACCI, Age adjusted Charlson Comorbidity Index*.

### The Predictive Effect of the ACCI Score on All-Cause Mortality and Hospital Readmission

**Figure 2:**
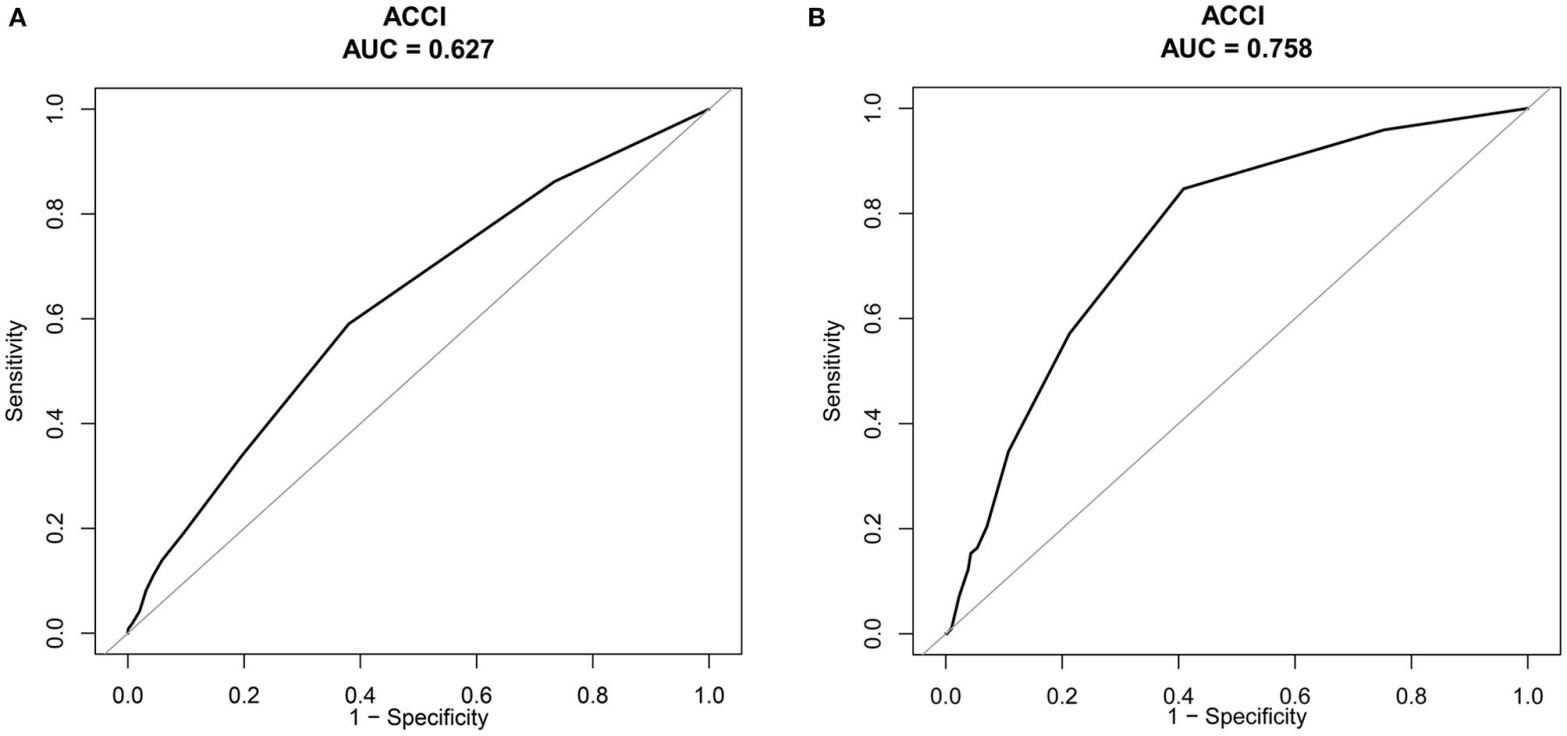
**(A)** Receiver operating characteristic (ROC) curves of 90-day hospital readmission. **(B)** Receiver operating characteristics (ROC) curves of ACCI for all-cause of 90-day mortality.

The outcomes of ROC curve analysis indicated that the discrimination performance of the ACCI was moderate for mortality (AUC: 0.758, 95% CI: 0.715–0.80; specificity = 0.591, sensitivity = 0.846) but poor for hospital readmission (AUC: 0.627, 95% CI: 0.605–0.648; specificity = 0.620; sensitivity = 0.590), as displayed in Figures 2A,B.

## Discussion

Our study found that an ACCI score and a high ACCI (≥5) were independent risk factors for all-cause mortality and hospital readmission among older Chinese surgical inpatients. To the best of our knowledge, this was the first large-scale, multicenter cohort study in China. The results stress the importance of assessing comorbidities before preoperative care, which could be an effective benchmark for predicting adverse outcomes for older surgical patients. Due to the high risk of older patients undergoing surgery, this study implies that surgical teams should closely work with corresponding medical specialists to manage the comorbidities of patients during the preoperative period. Appropriate comorbidity management programs should also be conducted for patients to reduce the rate of mortality and hospital readmission.

We found that the 90-day all-cause mortality rate was 2.51%, which is higher than previous research reporting 0.11% among 30,129 surgical patients who received orthopedic surgery in Japan (26). The main reason is that the age in our sample was older, and the overall mortality rate was included in the hospital and 90-day follow-up, whereas the Japanese study only calculated in-hospital mortality. Furthermore, the percentage of high ACCI was higher in our study than in the Japanese study. Another possible reason may be the discrepancy in surgical medicine and care between these two countries. A study conducted in the UK, a country with 487,197 older surgical patients, reported that the mortality rate at 28 days was 2.46%, which is similar to our study (27). In addition, a study including 1,004 patients who underwent fracture surgery reported that the in-hospital mortality rates at 30 days were 1.8 and 2.7%, respectively (28). Hence, preoperative assessment care, successful surgical procedures, and postoperative medical care were all priority actions to reduce the rate of mortality.

Our study revealed that every point increase in the ACCI score could increase the risk of hospital readmission by 18% for older surgical patients. Furthermore, surgical patients with a high ACCI score (≥5) had a 2.13-fold greater risk of hospital readmission than those with an ACCI score <5. Consistent with our findings, Becher et al. (29) discovered that elderly patients who underwent radical nephrectomy with a high CCI (≥4) had a greater risk of hospital readmission than those with a low CCI (40.01 vs. 9.09%, *P*<0.01). However, the age in our study was lower than that in Becher et al. (65 vs. 70 years or older). Many studies have shown that CCI can increase the risk of hospital readmission (12, 30–32). Thus, appropriate interventions should be implemented to reduce hospital readmissions among older surgical patients.

Several studies have explored the association between CCI or ACCI and mortality. In a large-scale cohort study including 487,197 surgical patients aged ≥50 years from New Wales hospitals, patients with a higher CCI (≥3) had an increased risk of 30-day mortality and 28-day hospital readmission, with ORs of 3.60 (95% CI: 3.40–3.82) and 2.02 (95% CI: 1.94–2.09), respectively, which is in line with our results (27). In addition, a population-based cohort study in China that included 210,450 patients who underwent hip arthroplasty surgery reported that patients with a CCI≥3 had an increased risk of mortality compared to those with a CCI of 0 (RR = 8.20, 95% CI = 6.5–10.4) (33). However, both of the abovementioned studies used the CCI tool to predict adverse outcomes. Since age is an important risk factor for postsurgical complications, combining comorbidities with age could be a more precise predictive tool. A study of 268 surgical patients performed by Qu et al. (34) found that the performance of ACCI was better than that of CCI for determining overall survival. Furthermore, in a study of 4,508 lung cancer patients after surgical operation, Yang el al. (15) revealed that ACCI scores were well associated with the risk of 3-year mortality (HR = 1.43, 95% CI: 1.08–1.90), which was similar to our outcomes. The average age in our study was older than that in Yang's research (72.46 ± 6.22 vs. 64.95 ± 11.15), but the share of those with a high ACCI (>5) in our study was similar in Yang's study (41.95% vs. 39.75%). In summary, ACCI was associated with an increased risk of all-cause mortality, which has been widely reported in different surgical patients. Thus, the ACCI score was an independent risk factor for all-cause mortality, which could be an appropriate prognostic indicator for clinical staff to identify high-risk groups and to implement suitable treatment.

Risk assessment tools for determining negative outcomes are numerous, including the modified Frailty Index (35), the American Society of Anesthesiologists' (ASA) physical status classification (36), the National Surgical Quality Improvement Program (NSQIP) (37), and the CCI. In our study, we found that the predictive ability was moderate, with an AUROC of 0.758 (95% CI: 0.715–0.80); the specificity and sensitivity were 0.591 and 0.846, respectively. Reis et al. (38) performed a study including 306 patients who underwent elective arterial vascular surgery, and the AUROC of the CCI was 0.732 (95% CI: 0.601–0.863), which was slightly lower than that in the study. Other studies have also applied the CCI for predicting mortality, with an AUROC ranging from 0.616 to 0.71 (28, 39). The CCI consists of medical conditions, infections, and other parameters for evaluating the end-organ dysfunction of oncologic history; it has been widely validated for estimating adverse outcomes in surgical patients (40, 41). Based on a poor AUROC, many studies have explored the new adjusted CCI for predicting mortality. The age-adjusted CCI was the most common predictive score applied in the clinical setting and was reported to boost performance (15, 18). Furthermore, some studies have used new methods, such as the novel machine learning technique, to develop a new surgical complexity score to predict all-cause mortality. Hyer et al. (39) found that a new surgical complexity score grounded in a novel machine learning technique had the best discrimination performance compared to the CCI and the Elixhauser Comorbidity Index (ECI). More studies comparing new predictive comorbidities should be conducted among older surgical patients in the future.

Our study has both strengths and limitations. First, to the best of our knowledge, this is the first study based on a large-scale, multicenter cohort focusing on older surgical patients with minimum selection bias. Second, we used comprehensive statistical analysis to calculate the independent effect of the ACCI for all-cause 90-day mortality and 90-day hospital readmission by fully adjusting for potential confounding factors and subgroup analysis. Third, we used a standard training program for the data collection procedure to ensure the quality of the data. However, we need to be cautious in interpreting our findings, with some limitations. First, our study was conducted among older Chinese surgical patients without validation in other countries, therefore limiting generalization. Second, data on some important geriatric syndromes (such as frailty, sarcopenia, malnutrition, etc.) were not collected at the design stage, which cannot be adjusted for these potential confounding factors. Third, data on some variables during the intraoperative period were not gathered, which may overestimate or underestimate the effect of the ACCI for predicting hospital readmission or mortality. Therefore, future studies should provide a comprehensive data set that includes the intraoperative period, which could offer more information for determining the association between ACCI and adverse outcomes. Fourth, this study did not collect data on adverse outcomes at 30 days. Future studies should also include 30-day mortality as an outcome since this is commonly accepted as a more concise postsurgical mortality timeframe. Fifth, this study failed to make comparisons with other risk score systems, such as the American College of Surgeons' NSQIP risk score and the ECI, which makes it unable to be used to understand predictive performance. Finally, we speculated that the indication for surgery might influence the risk of the outcome. However, we observed that the proportions of general anesthesia in the lower ACCL and high ACCL groups were 83.57% (1,897) and 81.47% (1,337), respectively, with no statistically significant difference (*p* = 0.088), which suggested that the type of surgery might be similar between these two groups. In addition, the proportions of general anesthesia in the death and survivor groups were 78.5% (77) and 82.80% (3,157), respectively, with no statistical significance (*p* = 0.275). Therefore, we might speculate that the type of surgery reflected by the variable of general anesthesia might not influence the outcome in our participants.

## Conclusion

In sum, a higher ACCL score was associated with a higher risk of mortality and hospital readmission, highlighting the predictive value of the ACCI for older Chinese surgical patients. This study suggests that the ACCI could assist clinical staff in guiding decision-making with patients and family members and in discussing the benefits and risks of surgical intervention during the process of informed consent.

## Data Availability Statement

The raw data supporting the conclusions of this article will be made available by the authors, without undue reservation.

## Ethics Statement

The Ethics Committee of Peking Union Medical Hospital approved this study (#S-700). The patients/participants provided their written informed consent to participate in this study.

## Author Contributions

X-JW is responsible for designing this study and initiating this concept. X-MZ was responsible for drafting the initial manuscript. CZ and X-MZ conducted all statistical analyses. JC, NG, JJ, Y-FM, and H-XB were responsible for collecting the raw data and organizing this project. All authors contributed to the article and approved the submitted version.

## Funding

The National Health and Family Planning Commission of China funded this study (Grant #201502017).

## Conflict of Interest

The authors declare that the research was conducted in the absence of any commercial or financial relationships that could be construed as a potential conflict of interest.

## Publisher's Note

All claims expressed in this article are solely those of the authors and do not necessarily represent those of their affiliated organizations, or those of the publisher, the editors and the reviewers. Any product that may be evaluated in this article, or claim that may be made by its manufacturer, is not guaranteed or endorsed by the publisher.
