# Supplementary material for: Effect of the Age-Adjusted Charlson Comorbidity Index on All-Cause Mortality and Readmission in Older Surgical Patients: A National Multicenter, Prospective Cohort Study
Source: Front Med (Lausanne). 2022 Jun 28;9:896451. doi: 10.3389/fmed.2022.896451 (PMC9274287; doi:10.3389/fmed.2022.896451)
Supplement: Supplementary file 1 [file Table_1.doc]

Supplemental Table1.a: The results of multicollinearity in regression Analysis for hospital readmission

| Variables | Tolerance | variance inflation factor |
| --- | --- | --- |
| Age  Gender  BMI  Coma  Mechanical Ventilation  Smoking  Urinary Catheters  Braden  Pneumonia  Hemoglobin  Leukomonocyte | 0.958 | 1.044 |
|  | 0.766 | 1.305 |
|  | 0.964 | 1.037 |
|  | 0.735 | 1.361 |
|  | 0.829 | 1.207 |
|  | 0.766 | 1.305 |
|  | 0.877 | 1.141 |
|  | 0.679 | 1.474 |
|  | 0.902 | 1.108 |
|  | 0.998 | 1.002 |
|  | 0.974 | 1.027 |

Supplemental Table1.b: The results of multicollinearity in regression Analysis for death

| Variables | Tolerance | variance inflation factor |
| --- | --- | --- |
| Age  Gender  BMI  Coma  Mechanical Ventilation  Smoking  Urinary Catheters  Braden  Pneumonia  Hemoglobin  Leukomonocyte | 0.958 | 1.044 |
|  | 0.766 | 1.305 |
|  | 0.964 | 1.037 |
|  | 0.756 | 1.323 |
|  | 0.829 | 1.207 |
|  | 0.766 | 1.305 |
|  | 0.877 | 1.141 |
|  | 0.679 | 1.474 |
|  | 0.902 | 1.108 |
|  | 0.998 | 1.002 |
|  | 0.974 | 1.027 |
